# Visual Attention and Strategic Sophistication in the Strategic Environment Effect: An Eye-Tracking Approach

**DOI:** 10.3390/jemr19040087

**Published:** 2026-08-13

**Authors:** Yukihiko Funaki, Yukio Koriyama, Ayano Nakagawa, Ali I. Ozkes

**Affiliations:** 1 Faculty of Political Science and Economics, Waseda University, Tokyo 169-8050, Japan; 2 CREST, Department of Economics, Ecole Polytechnique, IP Paris, 91120 Palaiseau, France; yukio.koriyama@polytechnique.edu; 3 Department of Technology Management for Innovation, Graduate School of Engineering, The University of Tokyo, Tokyo 113-8654, Japan; a.nakagawa@css.t.u-tokyo.ac.jp; 4 Department of Management, WU Vienna University of Economics and Business, 1020 Vienna, Austria; ali.ozkes@wu.ac.at; 5 GREDEG, Université Côte d’Azur, SKEMA Business School, 92150 Suresnes, France; 6 LAMSADE, Université Paris-Dauphine-PSL, 75116 Paris, France

**Keywords:** bounded rationality, cognitive engagement, eye-tracking, strategic environment, strategic sophistication

## Abstract

This study investigates how strategic environments shape a decision maker’s reasoning processes by incorporating visual attention and cognitive engagement. Using eye-tracking methods within experimental guessing games featuring interior equilibria, we analyze how strategic complementarity and substitutability alter choice behavior and cognitive engagement. We find that while choices diverge further from equilibrium under strategic complementarity, eye gaze metrics—specifically fixation and transition measures—are consistent with more intensive cognitive engagement under strategic substitutability. Furthermore, by using a novel approach to measure strategic sophistication, we find that individuals with higher strategic aptitude exhibit higher levels of visual engagement with the payoff table. As strategic sophistication increases, the two distinct dimensions of attention build up sequentially rather than together, with attention to an individual’s own strategies strengthening prior to attention to their opponents’. These findings indicate that visual attention is shaped systematically by the strategic environment and are consistent with a sequential organization of reasoning that may underlie the strategic environment effect.

## 1. Introduction

Strategic environment in games is characterized by the effect of a change in a player’s choice on the marginal payoff of the other player(s). In many games, whether the strategic environment exhibits complementarity or substitutability is observed to be an important factor in the aggregate outcomes. However, previous research is not conclusive regarding the mechanisms through which the strategic environment is affecting individuals’ decision-making processes. This paper sheds light on the ways the strategic environment affects decision-making by paying particular attention to strategic sophistication and examining the eye movements of experimental subjects.

Dubbed as the *strategic environment effect* (SEE), the observation that aggregate outcomes deviate more from the rational expectations equilibrium in games with strategic complementarity compared to strategic substitutability is prevalent. In oligopoly markets, for instance, it has long been observed that in Bertrand (price) competition there is more tacit collusion compared to Cournot (quantity) competition [1].The former is commonly modeled as a game with strategic complementarity, whereas the latter is generally characterized with strategic substitutability. SEE is also documented in guessing games (see [2], among others), which are the focus of this paper. These games represent a canonical model of strategic thinking dating back to the classical work by [3] and have been extensively studied in behavioral game theory [4].

Explanations for how this effect emerges are rather scant. In most studies, experiments entail repeated interactions and the explanations proposed for observed behavior in those instances (e.g., reciprocity as in [5], among others) are not portable to one-shot games. Ref. [6] established that in one-shot guessing games, distributions of hierarchical thinking levels remain constant across strategic environments, and given the same level distributions, hierarchical thinking induces the strategic environment effect necessarily. Hierarchical thinking models depict non-equilibrium thinking structurally such that players’ best responses rely on heterogeneous beliefs on others’ strategies, including naïve behavior and are implemented successfully toward explaining experimental observations from a wide range of games (see, *inter alia*, [7,8,9]). Given that hierarchical thinking levels are not affected by the strategic environment, this paper explores whether reasoning processes are influenced.

There are several steps when a decision maker (DM) makes a choice of their action facing a strategic interaction with others. For instance, after an initial information acquisition step, a reasoning stage in which the acquired information is processed (via learning, verifying, and checking) is necessary before applying an exercise of strategic thinking that eventually leads to decision making The DM may go through these steps recursively, that is, the DM may first acquire information before processing it, and then coming back to further information acquisition, before processing it, and so on. When the game is presented by a payoff table, tracking analyses based on eye or mouse movements have been successful in disentangling the complex structures behind the strategic decision making in small games, e.g., 2×2 or 3×3 games (see the literature review at the end of this section). In these studies, a fairly large part of the observed actions could be explained by specifying the DM’s norm, such as individual maximization (e.g., maxmax, maxave, or maxmin), as well as social preferences (e.g., efficiency, maxi-min, or envy, as in [10]), among others. When the game is small, detecting the DM’s norm in eye movements may be feasible as the steps of information acquisition and processing may be tractable given each norm. Once the patterns of eye gaze are detected in the data, it suffices to approximate the DM’s norm as the one that fits the observed patterns the best.

Our aim is to understand how the earlier stages of the reasoning process are shaped by the strategic environment. In this first eye-tracking study on the strategic environment effect, we focus on games that have relatively larger strategy space so that differing strategic environments can be visibly and distinguishably implemented. One may face several difficulties in extending findings from eye-tracking analyses to games with a larger number of pure strategies. First, figuring out the patterns may be more challenging, given the constraints due to the accuracy limits of the obtained eye-tracking data when the payoff table is displayed on the limited space of the screen. Second, when the strategy space is larger for both the DM and the opponents, the detection of the patterns in the above-mentioned steps of information acquisition and processing from the eye-tracking data may be fairly complex, which would render it difficult to estimate the DM’s norm. Third, even fixing a specific norm of the DM, its application to a larger game may require higher cognitive abilities, creating noise in the relationship between the eye gaze patterns and the norm, which makes the detection of the norm even harder.

Therefore, in our experimental design, we aimed at simplifying the design and payoff table so as to focus on the earlier stages of the reasoning process devoted to information processing, which can be captured by eye-tracking methods. In doing so, we consider a setup where the payoff structure is kept as simple as possible, with binary or ternary values and with best responses highlighted, so that the engagement of the DM with the payoff table can primarily be linked to the processing of information (on the payoff structure) rather than to information acquisition.

In our analysis, we investigate the impact of the strategic environment on reasoning processes via certain measures we obtain from eye movements that include characteristics such as the numbers or counts of *fixations* or *transitions* between pre-defined meaningful areas of the payoff table, as well as *fixation duration*. Concretely, fixation refers to the time interval where the gaze is held stable on a specific area, while fixation duration quantifies the time dedicated to that location. Transitions represent sequential movements of the fixations across distinct areas. We examine, furthermore, the interplay between strategic sophistication and reasoning. Although cognitive skills are found to be correlated with reaching certain outcomes (e.g., cooperative outcomes as in [11] and equilibrium outcomes as in [12]), the role of direct measures of ability relating to strategic sophistication in the context of SEE has not been explored. These abilities are known to be critically important in one-shot games or in the initial stages of repeated games [13,14].

In our data from the choices subjects made, we first and foremost confirm the strategic environment effect: choices, on average, diverge more from the NE in strategic complementarity. Next, we find that subjects’ eye movements are more intense under strategic substitutability. Specifically, their eye gaze involves more visits to the strategically critical regions of the payoff table and more frequent transitions between them, leading to a longer interaction time in the relevant areas in the payoff table. Finally, through a novel approach to the measurement of strategic sophistication utilizing item response theory, we are able to conclude that subjects with higher strategic aptitude are more engaged in their interactions with the specific areas in the payoff table, and this tendency (greater engagement of the high sophistication subjects) does not depend on the strategic environment. See Section 2 for a detailed account of our strategic sophistication measurement, which relies heavily on the explanatory power of hierarchical thinking models in the context of guessing games.

The remainder of the paper is organized as follows. This section is concluded with a literature review focusing on the strategic environment in different contexts including social dilemmas, market games, and guessing games, followed by eye-tracking methodology. Section 2 is devoted to a theoretical exposition of the strategic environment effect in the context of beauty contest games, and our experimental design and implemented procedures. Our hypotheses are stated in this section. Section 3 delivers experimental results and our central analysis, Section 4 is devoted to the discussion of our methods and results, and conclusions.

### Related Work

In his survey of the experimental literature on industrial organization [15] noted early on the by-then emerging evidence for the strategic environment effect. Refs. [16,17,18] had established theoretically how aggregate outcomes are affected by the strategic environment and co-existence of naïve and sophisticated players (see [19], as well). Ref. [1] concluded that in Bertrand (price) games with complementarity there is in general more collusion compared to Cournot (quantity) games with substitutability, ref. [5] established SEE via experiments that are designed to single out the strategic environment effect by eliminating certain previously identified confounding factors such as the market framing, externalities, incentives for cooperation, and so on, and [20] surveyed the oligopoly experiments from 2000 up until 2013. It should be noted, however, that when communication is allowed, the strategic environment effect is observed to disappear (see [21]).

Beyond the games with cooperation/collusion prospects, ref. [22] finds that asset prices in market games with strategic substitutability games converge quickly to their equilibrium value, whereas in games with strategic complementarity, it is more likely to observe large fluctuations in prices and deviations from the fundamental price (see also [23]). More recently, ref. [24] argues, in the context of a repeated pricing game with shocks (see also [25]), that convergence to equilibrium play might depend on the existence of players who are not performing well in processing information and forming expectations only in strategic substitutability.

Ref. [2], in the first ever study of the SEE in the context of guessing games, confirmed experimentally that the estimated hierarchical thinking heterogeneity is not affected by the strategic environment although SEE is observed. Ref. [6] proved theoretically that SEE is in fact implied by the equivalence of the distributions of hierarchical thinking levels and experimentally confirm that this is the case for both small and large numbers of players.

In previous research that implements eye-tracking methodology to uncover underlying processes of choice in game play, it is generally observed that subjects whose decisions indicate sophisticated reasoning are associated more strongly with eye gaze patterns that can be characterized by exploration and evaluation of both own and others’ payoffs compared to subjects whose decisions indicate more naïve reasoning (see, in particular, refs. [10,26,27,28,29,30,31] among others).

Ref. [32] demonstrates that the relation between lookup patterns and frequency of equilibrium play is a relation of causality. Ref. [33] finds that less sophisticated players change their gaze patterns towards the evaluation of others’ incentives, while cooperative players do not change their visual patterns. Ref. [34] finds that players exhibiting low cognitive reflection tend to pay less attention to relevant transitions between the counterpart’s payoffs. Ref. [35] identifies how the ways learning from previous games enhances strategic sophistication differ across subjects, by analyzing the eye movements.

In a study using neuroimaging techniques, ref. [36] observed more activity during choices in games with strategic complementarity compared to strategic substitutability in areas commonly associated with ‘intuition’ (e.g., insula) and with ‘deliberation’ (e.g., the dorsolateral prefrontal cortex).

## 2. Materials and Methods

### 2.1. The Games

We consider beauty contest games (BCGs) in which n∈N players simultaneously choose a number between 0 and 10. In order to study the strategic environment effect, we focus our analysis on the two variants of the game, BCG+ and BCG−. Player’s actions are strategic complements in the former, while they are strategic substitutes in the latter.

Let $x_{i}$ be the number chosen by player *i*. Each player’s objective is to choose a number closest to the target number, which is defined as a function of the average of the numbers chosen by all players except for the player. The target number for player i, $T_{+}^{i}$ (resp. $T_{-}^{i}$) in BCG+ (resp. BCG−) is defined by

$$T_{+}^{i}=2+\frac{2}{3}\frac{\sum_{j\neq i}x_{j}}{n-1}\;\mathrm{and}\;T_{-}^{i}=10-\frac{2}{3}\frac{\sum_{j\neq i}x_{j}}{n-1}.$$

In the case of a tie, a player is chosen randomly by a fair lottery to win the prize, and the players’ preferences are represented by a von Neumann–Morgenstern payoff function.

Under this specification, (i) both games have the same symmetric Nash equilibrium $x^{*}=6$, and (ii) the slopes of the best response function are equalized in absolute value between the two games. These properties allow us to extract the pure effect caused by the differences between strategic complementarity and substitutability, by changing only the sign of the best response function. For a more detailed discussion, we refer readers to Appendix B in [6].

### 2.2. Experimental Methodology

We implement a laboratory experiment consisting of four variants of the Beauty Contest Game: BCG+, BCG−, BCG1, and BCG2. In all treatments, the number of players is fixed at n=4.

The first two games, BCG+ and BCG−, constitute the main objects of analysis. BCG+ is designed to feature strategic complementarity, whereas BCG− features strategic substitutability. In these games, subjects are randomly matched with three other subjects and play each game once.

In the remaining two games, BCG1 and BCG2, subjects are asked to perform three separate *tasks* each. In each task, subjects choose an action against computer players whose strategies are generated according to distinct ways of strategic reasoning: level-1 (L1) [7,37,38], symmetric Nash equilibrium (NE), and inclusive cognitive hierarchy (ICH) [9]. Since each game comprises three tasks, each participant completes six tasks in total.

During all games and tasks, participants’ eye movements are continuously recorded using a remote eye-tracking system. This allows us to collect detailed measures of visual attention, including fixation locations and durations, as well as the number of visits to predefined areas of interest (AOIs). These data are used to characterize how patterns of visual attention vary across different strategic environments and decision processes.

Finally, using task-level performance indicators, we estimate a measure of individual strategic sophistication based on item response theory (IRT) [39]. This index captures heterogeneity in subjects’ ability to correctly solve tasks requiring different levels of strategic reasoning. We then relate this measure to observed eye-movement patterns in order to investigate how visual attention is associated with differences in strategic sophistication and underlying cognitive processes.

### 2.3. Logistics, Subjects, and Eye-Tracking Device

The experiment was conducted in July 2019 at Waseda University in Tokyo. We conducted 26 sessions with four undergraduate participants per session, yielding a total of 102 subjects (two no-shows), who came from a range of academic majors. Participants were recruited through Waseda University’s online recruitment system. The experiment was conducted in Japanese.

The laboratory consists of four visually isolated booths, each equipped with an eye-tracking device, a Tobii Technology T-120 eye tracker (Tobii Technology AB, Stockholm, Sweden), connected to a networked computer and a mouse. The system recorded gaze position at a sampling rate of 60 Hz (every 16.7 ms) with a spatial resolution of 1280 × 1024 pixels. For more detailed information on eye-tracking settings, see Appendix D. The booths are separated by partitions to prevent visual interaction among participants. Upon arrival, subjects were randomly assigned to one of the four booths and completed the experiment individually at their own pace.

The experiment consists of three parts. In Part 1, subjects played variants of the baseline Beauty Contest Game (BCG). In Part 2, participants completed several tasks using two variants of the Beauty Contest Game, BCG1 and BCG2. In Part 3, they played BCG+ and BCG−. Within each part, the order of games was randomized across subjects to mitigate order effects.

The experimental procedure proceeded as follows. First, written instructions were distributed, and participants read them at their own pace. Questions were allowed at any time. Second, each subject underwent individual calibration of the eye-tracking system to ensure accurate detection of gaze position. Third, subjects completed Part 1 while eye movements were continuously recorded. The interface displayed all relevant game parameters, including the target number and group composition.

After completion of Part 1, eye tracking was temporarily paused, and instructions for Parts 2 and 3 were distributed. Participants again read the instructions at their own pace and could ask clarifying questions. Eye tracking was then resumed, and subjects completed Parts 2 and 3 under identical recording conditions, with all game parameters displayed on-screen.

After completion of Part 3, eye tracking was stopped and participants completed a paper-based questionnaire.

The experiment lasted approximately 30 min on average. Subjects received a fixed participation fee of 1000 JPY (equivalent to 9.27 USD in July 2019), plus performance-based earnings paid after the session. Two games were randomly selected for payment: one from Part 1 and one from Part 3. In the selected Part 1 game, the winner received 500 JPY (4.63 USD). In the selected Part 3 game, payoffs were converted at a rate of 1 point = 500 JPY (4.63 USD). Total additional payments therefore ranged from 0 to 1000 JPY (9.27 USD). Average total earnings, including participation fee, were 1191 JPY (11.04 USD).

### 2.4. Experimental Design: The Three-Part Structure

The experiment consisted of three consecutive parts designed to progressively transition subjects from general game comprehension to the core strategic games under analysis. To minimize the learning effect and isolate the impact of the strategic environment, our experimental protocol separates task-familiarization and psychometric trait elicitation from our primary empirical environment.

While subjects completed all three parts, Part 3 serves as our primary object of analysis. Parts 1 and 2 were implemented as preparatory and calibration phases to guarantee that the eye-movement data recorded during the main tasks reflect strategic reasoning as directly and cleanly as possible.

#### 2.4.1. Preparatory Phases (Parts 1 and 2)

All subjects first completed Part 1 to ensure that they were thoroughly accustomed to the *p*-beauty contest games. They played a series of seven standard *p*-beauty contest games, choosing an integer between 1 and 100 under varying parameters and against both human and computerized opponents. This stage allowed subjects to familiarize themselves with the general choice entry system and baseline strategic interaction.

Following this baseline adaptation, subjects entered Part 2, which was designed to elicit a measure of individual strategic sophistication. Subjects completed six discrete decision tasks using two matrix games (BCG1 and BCG2). The other players’ behaviors were fully determined by computer players, systematically varied to correspond to specific behavioral benchmarks: Level-1 reasoning (L1), a symmetric Nash equilibrium (NE), and the inclusive cognitive hierarchy (ICH) model [9].

Importantly, no feedback was provided during Part 1 and 2 to prevent ongoing learning effects. The choices elicited in this phase were subsequently used to estimate individual latent trait scores via an IRT, mapping each participant into a specific strategic sophistication stratum (High, Middle, or Low IRT group).

An exhaustive description of the procedural parameters, game variants, and interface for Parts 1 and 2 are relegated to Appendix C.

#### 2.4.2. The Core Games (Part 3)

Part 3 constitutes the core of our experimental design and yields the primary dataset for our empirical analysis. In this phase, we implemented a within-subject design to observe how visual processing adapts between two fundamentally distinct strategic environments: a game characterized by strategic complementarities (BCG+) and a game characterized by strategic substitutability (BCG−). The order of the two games was randomized across sessions to control for potential order effects.

As in the preparatory tasks of Part 2, the pure-strategy set consists of the integers from 0 to 10, corresponding to a discrete version of the game described in Section 2.1. The games are simplified in several ways from their standard counterparts with 100 pure strategies, to reduce the extraneous cognitive burden. Specifically, each player’s payoff depends solely on their own strategy and the rounded average of the other three players’ strategies.

**Figure 1:**
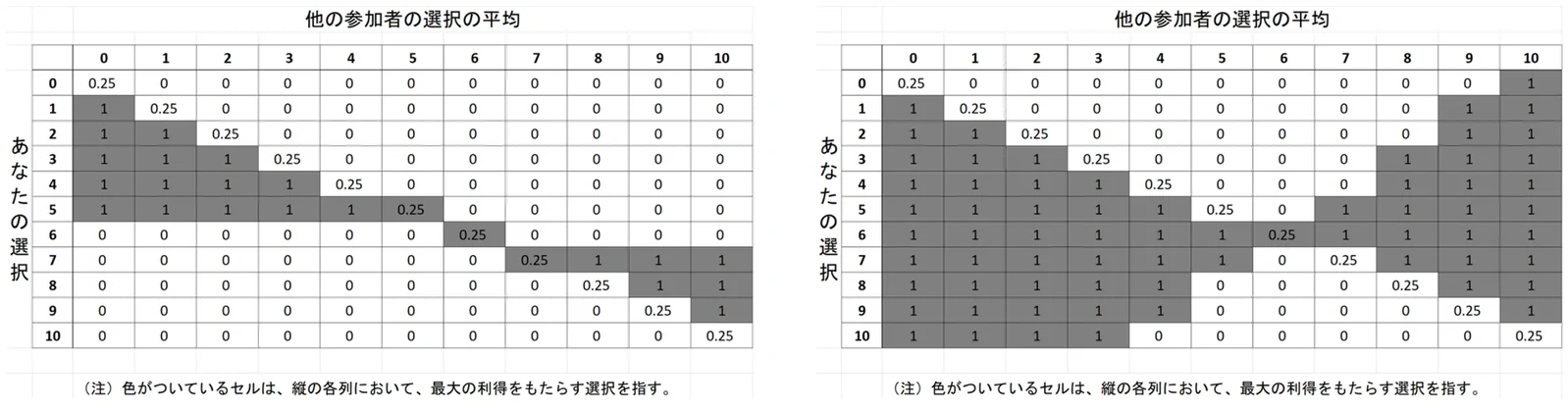
Payoff tables displayed on the screen in Part 3. Best-response regions are shaded. (**Left**): BCG+; (**Right**): BCG−. The left axis label in Japanese translates to “Your choice”; the top label translates to “Average choice of the other participants”; and the bottom note indicates that “The shaded cells indicate the choice that yields the highest payoff within each column”.

Furthermore, to minimize the purely computational burden of identifying optimal strategies, the best-response regions are explicitly highlighted by shading in the payoff tables displayed on the screen (Figure 1). This explicit design choice ensures that the recorded eye movements capture the underlying strategic reasoning patterns directly, rather than the information acquisition of the game structure by going through basic arithmetic computation.

Unlike the computerized setups in the calibration phases, Part 3 features no automated players. Each subject played both BCG+ and BCG− exactly once, and payoffs were determined entirely by the actual choices of the human participants in the other booths within the session. The payoff numbers (0, 0.25, or 1) indicate the points received by the subject, where 0.25 corresponds to the symmetric case where all players select the identical integer and split the prize with equal probability.

Eye movements were recorded throughout the decision period; the game ended at the moment the subject submitted their choice, so all recorded gaze data necessarily precede the decision.

### 2.5. Observed Variables, AOIs

Our eye-tracking analysis focuses on fixations, defined as brief periods during which the eyes remain relatively stable and visual information is actively acquired from a specific location in the visual field. Fixations are identified using the following parameter value thresholds: a minimum duration threshold of 60 ms, a maximum gap length of 75 ms, a velocity threshold of 30 degrees per second, a maximum time between fixations of 75 ms, and a maximum angle between fixations of 0.5 degrees. For each game and task, we record the position and duration of fixations sequentially from the moment the payoff table appears on the screen until the subject makes a decision.

To structure the analysis, we define several areas of interest (AOIs) corresponding to distinct regions of the display, and characterize for each AOI the number of fixations it attracts (hits) as well as the transition dynamics across AOIs. Each AOI hit is a binary variable equal to 1 when a fixation is recorded in that AOI and 0 otherwise. Since each hit is associated with a specific fixation, it inherits the corresponding timestamp and duration, enabling a dynamic analysis of how subjects’ gaze transitions across AOIs over time.

These variables are jointly analyzed together with the choices made by each subject in the game.

### 2.6. Estimation of Strategic Sophistication via Item Response Theory

To establish a robust, continuous measure of strategic sophistication, we employ an item response theory framework. Broadly defined, IRT is a prominent psychometric and statistical paradigm used to evaluate latent traits, such as cognitive ability, strategic aptitude, or sophistication that cannot be directly observed [40]. Unlike raw sum scores, which treat all test items equally, IRT modeling simultaneously estimates the latent ability of each subject and the structural characteristics of each task, such as its difficulty and discriminating power. This approach explicitly accounts for the fact that solving a highly complex strategic problem provides stronger empirical evidence of a subject’s strategic sophistication than correctly answering an intuitive or trivial one.

In our implementation, the two-parameter logistic (2PL) IRT scores are estimated via Marginal Maximum Likelihood approach in Stata 19 utilizing the six tasks completed by all subjects in Part 2. Because each of these tasks requires subjects to select a best response against three computer-automated players whose underlying strategy profiles are fully and explicitly specified, every task possesses a well-defined set of correct choices. We pool the binary performance records (correct versus incorrect responses) across the entire subject pool to parametrically estimate the structural weight and difficulty parameters of each task. Using these pooled estimates, each subject’s latent strategic sophistication score was extracted as empirical Bayes means (expected a posteriori estimates). This optimization assigns higher analytical weight to more demanding tasks, thereby yielding a continuous standardized latent measure of strategic sophistication for our subsequent non-linear behavioral analysis.

We note that our initial sample size is relatively small for complex item response theory estimations. However, while models estimating guessing parameters (e.g., 3PL) typically require vast sample sizes, psychometric literature establishes that sample sizes of 100 to 200 are generally sufficient for stable parameter and latent trait estimation in simple 2PL models, particularly when the test contains a small number of items and the primary analytical goal is ranking participant-level abilities [41,42]. While treating estimated IRT scores as continuous predictors without fully incorporating latent measurement error represents a methodological limitation, the overall psychometric precision of our IRT model, combined with the high statistical significance of our main findings, renders prospective attenuation bias negligible.

### 2.7. Hypotheses

We organize our analysis around three sets of hypotheses. The first concerns the strategic environment effect (SEE), testing whether this effect extends to our experiment with the beauty contest game.

**Hypothesis** **1.** *Subjects’ choices deviate further from the Nash equilibrium in games with strategic complementarity than in those with strategic substitutability.*

The next set of hypotheses aims to characterize the information-processing patterns accompanying the SEE. We conjecture that strategic substitutability demands a more engaged reasoning process, and we measure this engagement along two dimensions: *attentional intensity* and *reasoning complexity*.

Attentional intensity captures how intensely subjects focus on strategically critical regions of the payoff table. We measure it by visit counts to areas of interest (AOIs) and total fixation duration in the best-response area.

Reasoning complexity captures how extensively subjects navigate the reasoning process. We consider two constituent aspects of strategic thinking: best-response reasoning, in which the subject evaluates her payoffs holding the opponent’s strategy fixed, and belief formation, in which the subject scans the payoff table holding her own strategy fixed. They are proxied by vertical and horizontal fixation transitions in the payoff table, respectively, with total transition counts serving as a summary metric of overall reasoning complexity.

**Hypothesis** **2.** *Attentional intensity is higher in games with strategic substitutability than in those with strategic complementarity.*

**Hypothesis** **3.** *Reasoning complexity is higher in games with strategic substitutability than in those with strategic complementarity.*

Together, Hypotheses 2 and 3 outline a parallel process tracing of the SEE. By examining whether subjects adjust their attentional intensity and reasoning complexity across payoff structures, we trace how information-processing patterns systematically shift alongside choice behavior under substitutability versus complementarity.

The next set of hypotheses concerns the relationship between subjects’ engagement in strategic thinking and their degree of strategic sophistication. Our central conjecture, in line with the findings in the literature, is that subjects with higher strategic sophistication engage more intensely in strategic thinking [8,26]. We examine this conjecture through the lens of the two dimensions introduced above, belief formation and best-response reasoning, and test how each relates to the subject’s degree of strategic sophistication.

**Hypothesis** **4.** *Both the degree of best-response reasoning, as proxied by own-strategy visual attention, and the degree of belief formation, as proxied by opponent-strategy visual attention, are positively correlated with subjects’ strategic sophistication.*

**Hypothesis** **5.** *At the lowest levels of sophistication, both belief formation and best-response reasoning are present but limited. As the sophistication level increases, best-response reasoning intensifies first, while belief formation remains low. As sophistication increases further, belief formation begins to rise while best-response reasoning remains elevated. At the highest levels of sophistication, both belief formation and best-response reasoning are elevated.*

Hypothesis 4 establishes that strategic sophistication is associated with greater engagement along both dimensions. Hypothesis 5 refines this by asking whether the two dimensions scale with sophistication at the same rate. By examining how specific eye-movement measures vary with sophistication, we aim to shed light on its relationship with strategic thinking.

Collectively, the hypotheses outlined above pursue a broader goal, namely, to decompose the complex process of strategic thinking into tractable pieces, and in doing so, to reveal the underlying structure of strategic reasoning.

## 3. Results

The results are organized around the five hypotheses stated in Section 2.7: Result 1 corresponds to Hypothesis 1, and so on. The remaining analyses reported within each subsection provide descriptive details, rather than constituting independent tests.

### 3.1. Strategic Environment Effect

**Figure 2:**
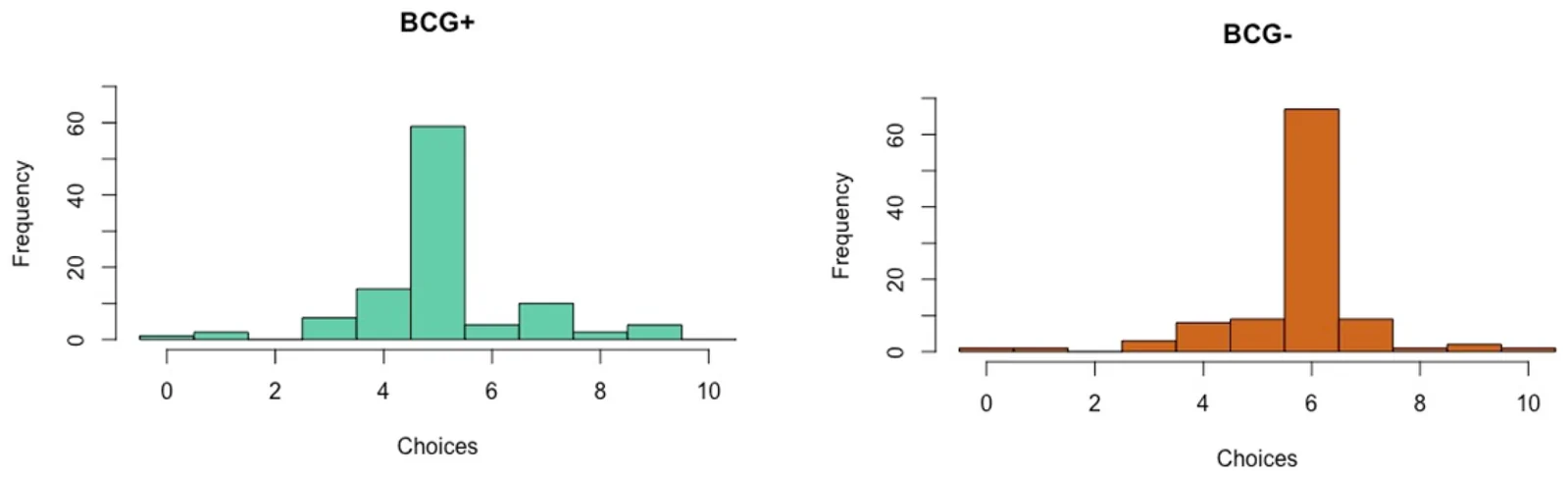
Histogram of subjects’ chosen actions (0–10) in BCG+ (**left**) and BCG− (**right**), N=86.

In our experiment, SEE corresponds to the observation that actions taken by players deviate more from the symmetric Nash equilibrium in BCG+ than in BCG−. Figure 2 shows the distribution of strategies chosen by 86 subjects in each game. Out of 102 participants, 16 are excluded from the analysis. A total of 14 subjects whose total gaze rate falls below 50%, or whose gaze rate in either game falls below 10%, are excluded as these indicate insufficiently reliable eye-tracking data. Additionally, two subjects are excluded after inadvertently skipping the entire BCG− game with a mistaken click. Further information on exclusion criteria is provided in Appendix D. The average strategy is 4.98 in BCG+ and 5.74 in BCG−, with standard deviations of 1.50 and 1.19, respectively. The distributions suggest that choices in BCG+ are more dispersed around the equilibrium, consistent with the presence of the SEE.

To assess the SEE more formally, we apply the hit rate area analysis axiomatized by [43]. This metric, known as Selten’s measure of predictive success (m=r−a), quantifies how well a theoretical model predicts empirical behavior by adjusting for the precision of the prediction. Here, the hit rate (*r*) represents the proportion of subjects’ choices that fall within a defined set of the Nash equilibrium prediction, while the area (*a*) captures the relative size of the prediction set. By evaluating r−a, this measure rewards empirical accuracy while penalizing overly broad or non-specific theoretical predictions.

**Result** **1.** *We observe a strategic environment effect: predictive success of the Nash equilibrium is 41.3% in BCG+ and 58.4% in BCG−, indicating that participants deviate more frequently from the equilibrium under strategic complementarity than under strategic substitutability. The Hodges–Lehmann estimate of the paired difference in absolute deviation from the Nash equilibrium between BCG+ and BCG− is 1.0 (95% CI: [1.0, 1.5]; Wilcoxon signed-rank test, W=337.0, p < 0.001).*

### 3.2. Eye Gaze Patterns

To understand how the SEE emerges, we examine subjects’ eye gaze patterns in detail, comparing them across the two strategic environments.

#### 3.2.1. Fixation Count

Table 1 reports the average number of fixations on the screen. Overall, fixation counts do not differ significantly between BCG+ and BCG− (Wilcoxon signed-rank test, p=0.7978). The table further breaks down fixation counts by three groups of participants categorized by strategic sophistication, as measured by the IRT score. The three groups differ in size because the IRT score, generated from six tasks, takes discrete values, and multiple subjects may fall on the boundaries. The Low, Mid, and High groups correspond to IRT score intervals of [−1.425,−0.357], [−0.302,0.615], and [0.647,1.270], respectively. Results are robust to the choice of thresholds. More sophisticated participants tend to make more fixations overall; however, within each group, fixation counts are similar across the two games, with no statistically significant differences.

#### 3.2.2. Visit Count and Fixation Duration: Attentional Intensity

The SEE is often attributed to the structure of the best-response function, which, in games with strategic substitutability, induces best responses to switch to the opposite side of the symmetric Nash equilibrium in response to others’ deviations [6]. This generates a two-sided pattern of deviations as best responses move across the equilibrium. By contrast, in games with strategic complementarity, best responses remain on the same side of the equilibrium, and thus do not exhibit such switching behavior (see Section 2.1).

**Figure 3:**
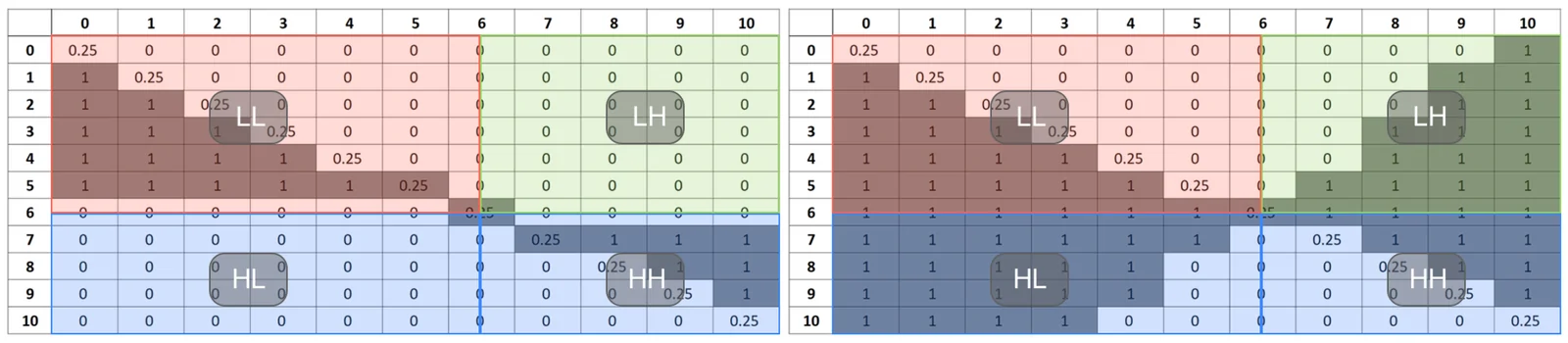
Four areas of interest in the payoff matrix: LL, LH, HL, HH, where the first letter is the subject’s own strategy (Low/High relative to $x^{*}=6$) and the second is the opponents’ average strategy. (**Left**): BCG+; (**Right**): BCG−.

To examine whether these structural differences are reflected in eye movements, we divide the strategy space into two intervals, below and above the symmetric Nash equilibrium $x^{*}=6$, and combine participants’ own and opponents’ strategies to define four areas of interest (AOIs) in the payoff matrix: {LL,LH,HL,HH} (Figure 3), where the first letter indicates the participant’s strategy and the second the opponents’.

Table 2 reports visit counts and fixation durations across the four AOIs. Overall visit counts are significantly higher in BCG− than in BCG+ (p=0.008), while total fixation counts show no significant difference (Table 1). This indicates that participants shift their gaze more frequently between regions under strategic substitutability, reflecting a more dynamic scanning of the payoff table without a corresponding increase in overall looking time.

These patterns closely mirror the structure of the best response function. In BCG−, best responses cross the equilibrium, requiring participants to compare strategies on both sides, whereas in BCG+, best responses remain on the same side. This structural difference is reflected in eye movements: gaze transitions are more frequent where best responses span multiple AOIs, while overall fixation time remains comparable across games. This alignment between gaze patterns and task structure is consistent with evidence that eye movements reflect underlying cognitive processing [44,45].

It is worth clarifying the distinction between the two eye-movement measures reported above. Fixation count measures how many times the eye pauses anywhere on the screen, capturing the overall quantity of visual attention without regard to where or how purposefully subjects look. Visit count, by contrast, measures how many times a subject returns to a specific AOI, capturing repeated and deliberate engagement with strategically meaningful regions of the payoff table. As we saw above in Table 1, fixation counts do not differ significantly between BCG+ and BCG−, indicating that subjects do not differ in how much they look overall. Visit counts, however, are significantly higher in BCG−, suggesting that subjects return more deliberately to specific regions under strategic substitutability, a pattern consistent with greater attentional intensity rather than merely greater visual activity.

Examining the four AOIs individually, visit counts and fixation durations are both significantly higher in LH and HL for BCG−, precisely the regions where the best response areas differ most between the two games. By contrast, LL and HH show little difference across games.

**Result** **2.** *Taken together, the evidence in Table 1 and Table 2 indicates that subjects’ gaze patterns, both in the frequency of visits and the time spent within each region, closely track the structure of the best response function, underscoring the link between strategic task structure and cognitive engagement. These results support Hypothesis 2.*

A potential concern is whether environment-dependent differences in transition and visit counts could simply reflect differences in total duration or the number of available fixation opportunities. However, a paired Wilcoxon test revealed no significant difference in total fixation duration between BCG+ and BCG− (W=2238.0,p=0.876; median: 6871.00 ms vs. 6889.00 ms). Because decision time was balanced across strategic environments, the observed variations in raw transition and visit counts reflect structural alterations in information acquisition and gaze organization, rather than artifacts of longer processing windows.

#### 3.2.3. Fixation Dynamics: Reasoning Complexity

We now turn to the dynamics of fixations. For each subject and each game, we examine consecutive pairs of fixations and analyze how gaze moves, or remains across AOIs. Table 3 reports total fixation transition counts across the four AOIs in the payoff table, together with two subsets of particular theoretical interest: vertical and horizontal transitions. Total transitions capture overall navigation across AOIs in the payoff table. Vertical transitions, defined as transitions between LL and HL, and between LH and HH, capture subjects’ best-response reasoning, reflecting changes in the subject’s own strategy while holding the opponent’s strategy fixed. Horizontal transitions, defined as transitions between LL and LH, and between HL and HH, capture belief formation, reflecting changes in the opponent’s strategy while holding the subject’s own strategy fixed.

**Result** **3.** *All three measures are significantly higher in BCG− than in BCG+, indicating that subjects navigate the payoff table more extensively under strategic substitutability. This is consistent with a higher degree of reasoning complexity in BCG−, as formulated in Hypothesis 3.*

This finding accords with the standard explanation of the SEE. In BCG−, the best response to opponents’ strategies with bounded rationality lies on the opposite side of the Nash equilibrium, whereas in BCG+, it remains on the same side. As a result, iterative best responses are expected to cross the Nash equilibrium more frequently under strategic substitutability than under strategic complementarity. Under the assumption that fixation transitions reflect subjects’ iterative best-response reasoning, the observed pattern of eye movements is consistent with this mechanism, offering a possible interpretation of the SEE.

**Figure 4:**
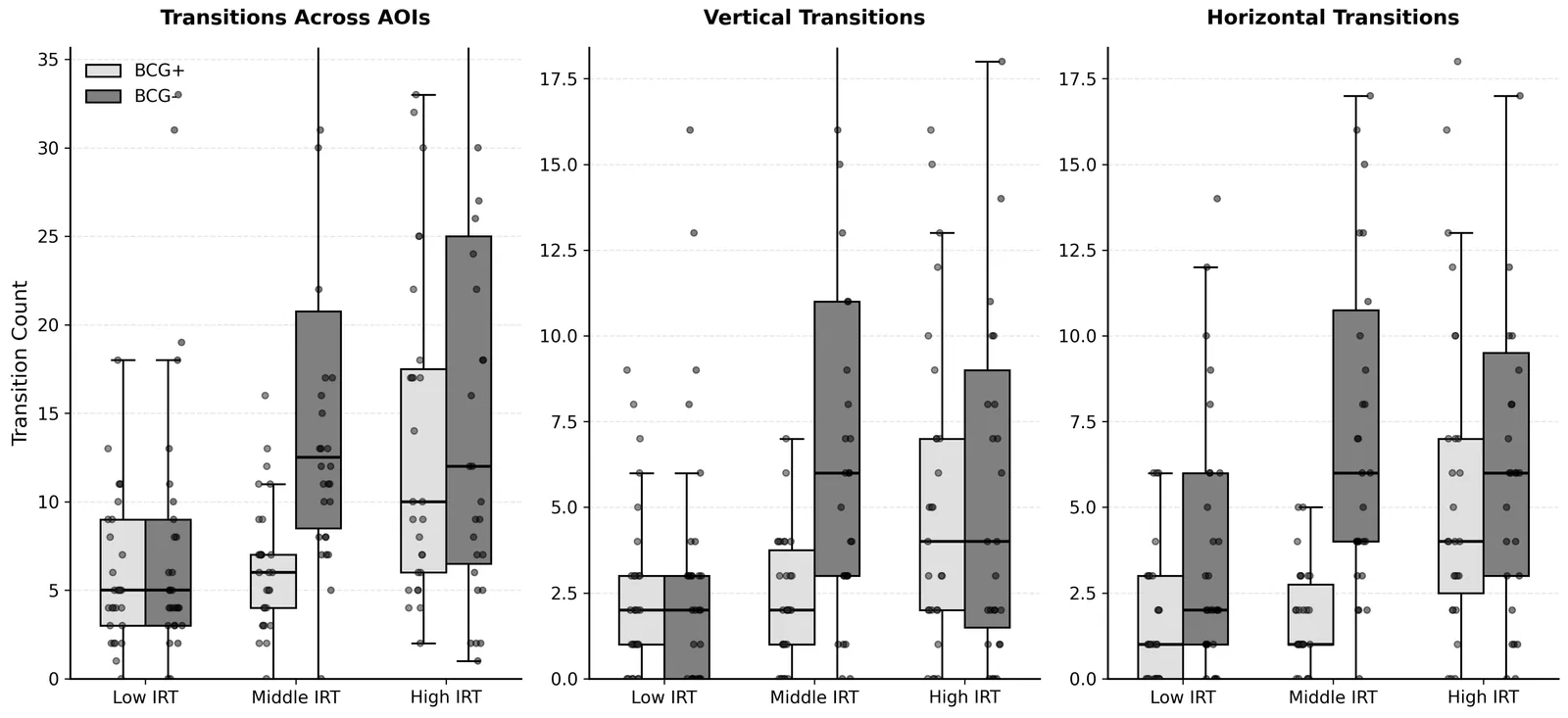
Transition comparison by IRT tertiles.

Figure 4 compares transition counts across the three IRT tertiles for BCG+ and BCG−. Several patterns emerge. First, the transition counts generally show an upward trend with respect to IRT scores, but exhibit a non-linear or non-monotonic pattern. The Spearman’s rank correlation test reveals a highly consistent pattern: the IRT score is significantly and positively correlated with all three transition metrics across both strategic environments (*p* < 0.001 for all specifications, except for vertical transitions under BCG− with p=0.051). This is consistent with Hypothesis 4, which predicts a positive relationship between subjects’ strategic sophistication and their engagement in the reasoning process.

Second, all three measures, *total*, *vertical*, and *horizontal* transition counts, are significantly higher in BCG− than in BCG+ across all tertiles (Wilcoxon signed-rank test, *p* < 0.001, in Table 3), confirming that the higher degree of reasoning complexity under strategic substitutability is robust across all levels of strategic sophistication.

Third, and most strikingly, this increase in the number of transitions under strategic substitutability (BCG−) is most pronounced among subjects in the middle tertile (Figure 4). This non-linear pattern is confirmed by a non-parametric between-group comparison of within-person change scores analysis. Specifically, we define an individual-level score (Δ=[BCG−]−[BCG+]) for each visual metric and compare these delta values across the three IRT strata using two-sided Mann–Whitney *U* tests. For total visual exploration (*total transitions*), the Middle IRT group exhibits a remarkable difference (Δ=13.23, SD =18.98), which is statistically significantly larger than the modest differences observed in both the High group (Δ=4.30, SD =14.89; Mann–Whitney *U* test, p=0.022) and the Low group (Δ=1.86, SD =7.29; *p* < 0.001).

Crucially, this non-linear expansion among the Middle group emerges consistently across all of our metrics. Regarding best-response reasoning (*vertical transitions*), the Middle group’s delta (Δ=6.40, SD =8.76) is significantly larger than that of the High group (Δ=1.78, SD =8.80; p=0.010) and the Low group (Δ=0.69, SD =3.52; *p* < 0.001). Similarly, for belief formation (*horizontal transitions*), the Middle group’s increase (Δ=7.07, SD =9.01) is significantly larger compared to both the High group (Δ=2.89, SD =6.76; p=0.014) and the Low group (Δ=2.14, SD =3.83; p=0.002).

These results demonstrate that strategic substitutability does not scale visual processing across all populations uniformly. Rather, it affects most substantially the middle-tier individuals, while the top tier exhibits a sustained high level of transition counts and the bottom tier shows consistently limited visual exploration across both strategic environments.

To complement Results 2 and 3, we estimated both level and within-person change models examining the link between our process measures and the behavioral outcome (ChoiceDist, the absolute distance from the Nash equilibrium; see Appendix A). Across both specifications, visual search metrics show no significant effect on choice behavior, supporting the view that the strategic environment induces distinct, parallel adaptations in decision outcomes and gaze patterns.

#### 3.2.4. Strategic Sophistication and Engagement in Reasoning Process

To elucidate the underlying cognitive mechanism driving the SEE, we further investigate how a subject’s engagement in the reasoning process varies according to their degree of strategic sophistication. Grounded in the hypothesis that highly sophisticated individuals exhibit more structured and dynamic visual search patterns in strategic thinking, we explore subjects’ visual exploration patterns in relation to their IRT scores. Specifically, we scrutinize how this relationship differs under strategic complementarity versus strategic substitutability.

Table 4 shows the estimation results of linear mixed-effects regression models. We evaluated two complementary dependent variables capturing distinct dimensions of visual processing: total fixation count (frequency) and total fixation duration (processing time). To account for repeated measurements (BCG+ and BCG−) from the same participant in our within-subject design, subject-specific random intercepts were estimated using a mixed-effects regression model.

The empirical results reveal a remarkably consistent and robust behavioral pattern across both metrics. First, we identified a highly significant and positive effect of trait-level strategic sophistication. Higher IRT scores predicted a substantial increase in both fixation count (*p* < 0.001) and total fixation duration (p=0.001). This indicates that individuals with higher strategic sophistication engage in more active and dynamic strategic thinking, maintaining a distinct pattern of visual engagement across both strategic environments. Conversely, the effect of the strategic environment (dummy variable *BCG*−) did not show statistical significance in either the count model (p=0.457) or the duration model (p=0.330).

Crucially, the interaction term (IRTscore × *BCG*−) does not reach statistical significance in either specification (β=−0.620, SE=7.472, 95%CI=[−15.265,14.025], p=0.934 for count; β=−0.228, SE=1.567, 95%CI=[−3.300,2.844], p=0.884 for duration). We therefore find no statistically detectable moderation of the sophistication effect by the strategic environment. While a non-significant interaction with bounded confidence intervals does not establish strict equivalence or invariance, our data provide no empirical evidence that the relationship between sophistication and visual attention differs substantially across strategic environments. In terms of model parsimony, the data do not support including a moderation pathway, suggesting that the association between trait-level sophistication and information processing operates similarly across both environmental contexts in our sample.

#### 3.2.5. Best Response, Belief Formation, and Strategic Sophistication

To further investigate the underlying cognitive mechanisms driving the SEE, this section explores the critical linkages between a player’s strategic sophistication and two core pillars of strategic reasoning: the formulation of a best-response strategy and the formation of beliefs concerning opponents’ behaviors. While standard analysis often focuses on the payoff values themselves, strategic thinking fundamentally requires a player to deliberate over both their own choice set and the potential choices of their rivals. Therefore, when facing a payoff matrix, a player does not merely aggregate numerical payoff information; they must actively process and evaluate the pure strategies available to themselves and their opponents. Consequently, we can expect that a player’s eye-gaze dynamics contain rich information reflecting such strategic deliberation process, moving beyond simple arithmetic payoff considerations.

**Figure 5:**
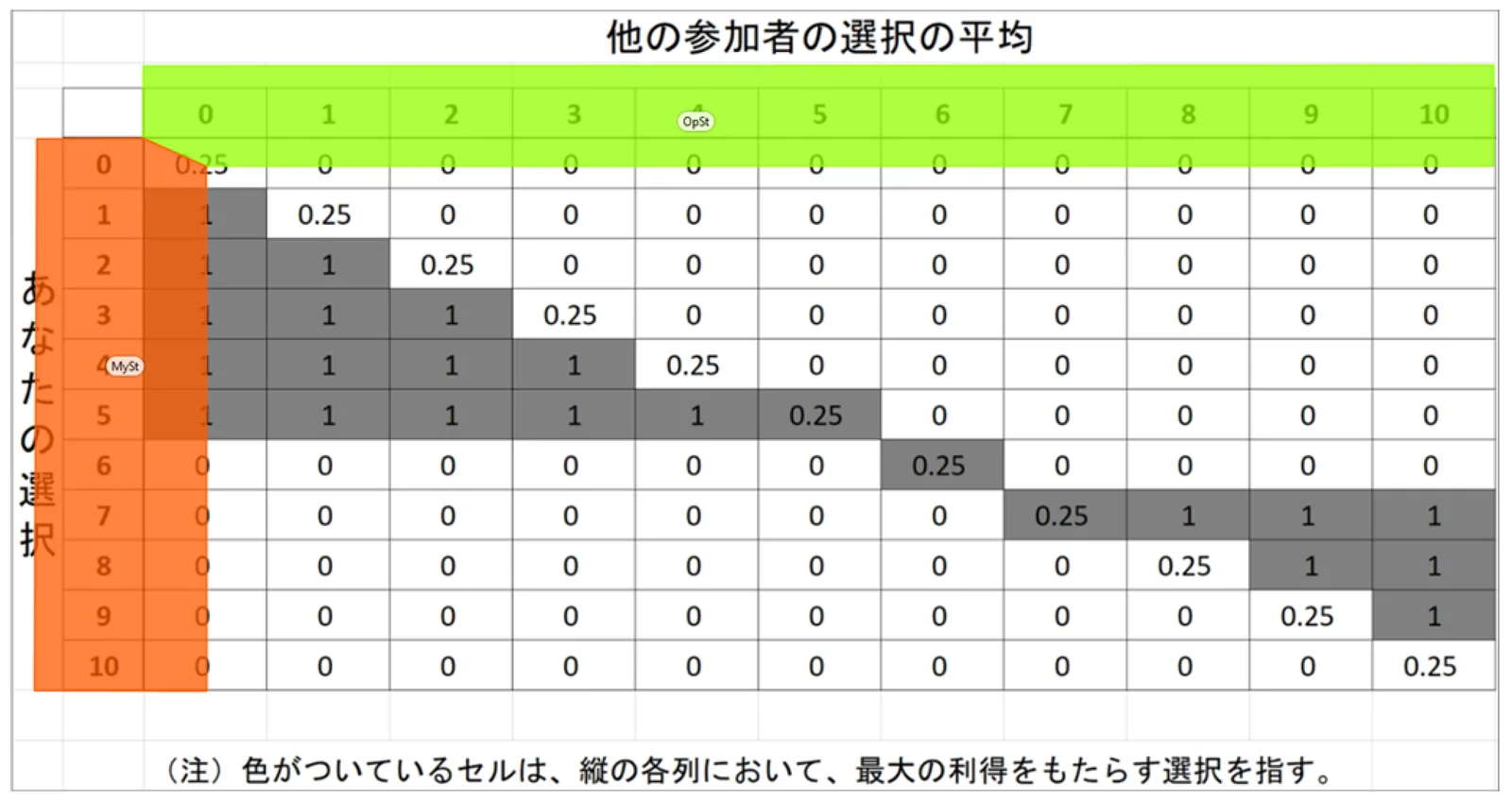
Definition of areas of interest (AOIs) for strategy labels. Note: *MySt* (orange) and *OpSt* (green) define the AOIs covering the labels of the subject’s own pure strategies and their opponents’ strategies, respectively, including a standard buffer zone.

To capture this specific cognitive dimension, we define two unique AOIs that target the structural labels of the matrix rather than the numerical payoff cells: the *my strategies* (*MySt*) area and the *opponent strategies* (*OpSt*) area (Figure 5). Focusing on these strategy labels is theoretically justified by the established eye-mind hypothesis, which posits that visual fixation closely mirrors immediate cognitive processing [44]. When a subject’s eye gaze dwells within the area of a specific strategy label, it is reasonable to infer that they are actively contemplating that particular strategic option or forming a belief about their opponent’s choice. By focusing our analysis on subjects’ eye-gaze dynamics over these strategy labels, we aim to isolate the explicit reasoning pathways through which strategic sophistication shapes information processing in complex environments.

The empirical results strongly corroborate our core hypotheses regarding the positive correlation between subjects’ strategic sophistication and engagements in strategic thinking.

First, we address Hypothesis 4, which posits a positive baseline relationship between subjects’ strategic sophistication and their engagement in both best-response reasoning and belief formation. To empirically test this assumption, we evaluate the correlations between subjects’ strategic sophistication (IRT scores) and their visual exploration intensity on the respective strategy labels, as tabulated in Table 5. The statistical outcomes consistently reveal that both own-strategy (*MySt*) and opponent-strategy (*OpSt*) fixation counts exhibit significant and positive correlations with strategic sophistication across both environmental regimes. Specifically, under strategic complementarity (BCG+), higher strategic sophistication is robustly associated with increased attention to both choice sets, with both parametric Pearson and non-parametric Spearman tests yielding high statistical significance. This positive association cleanly extends to the strategic substitutability environment (BCG−); the correlations remain highly significant for *MySt* across both metrics and for *OpSt* under the parametric assumption, while the non-parametric baseline for *OpSt* remains marginally significant.

**Result** **4.** *Visual proxies for best-response reasoning (MySt) are consistently and positively correlated with strategic sophistication across both environmental regimes. In contrast, the association for belief formation (OpSt) is environment-dependent, showing a robust positive correlation under BCG+, but only a marginal trend under BCG−. This indicates that while sophisticated players universally intensify their processing of own-payoff structures, their engagement with opponent dimensions is modulated by the underlying strategic environment.*

**Figure 6:**
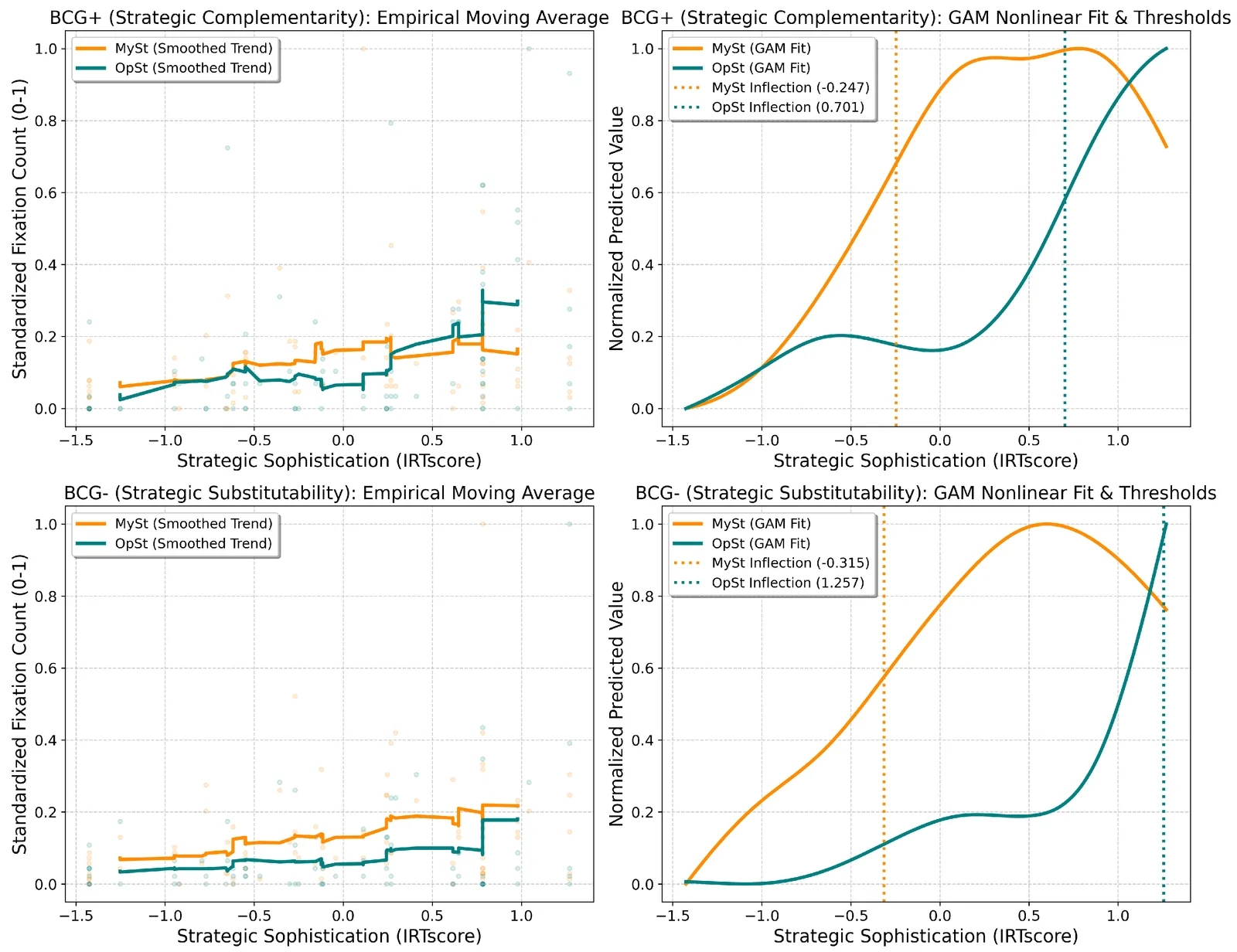
Empirical trends and non-linear generalized additive model (GAM) estimations of visual strategic attention. The left panels display the empirical moving averages of standardized fixation counts for own-strategy (*MySt*) and opponent-strategy (*OpSt*) labels. The right panels show the GAM fits along with the estimated inflection thresholds (maximum acceleration points) demarcated by vertical dotted lines for both the strategic complementarity (BCG+) and strategic substitutability (BCG−) environments.

Second, we scrutinize Hypothesis 5, which details a non-linear progression in how these two cognitive components intensify as strategic sophistication increases. To rigorously test this predicted sequential onset and asymmetry, we estimated generalized additive models (GAMs) [46,47] to identify the *inflection thresholds*, defined as the specific IRT scores where visual attention exhibits the maximum rate of increase (the steepest slope). This threshold analysis suggests a sequential hierarchy in strategic reasoning. These non-linear estimation results are presented in Figure 6. Under strategic complementarity (BCG+), the visual processing of own-strategy labels increases at a significantly lower level of sophistication (*MySt* inflection IRT=−0.247) than that of the opponents’ choices (*OpSt* inflection IRT=0.701), yielding a substantial asymmetry delta of 0.948. Remarkably, this sequential onset becomes even more pronounced under strategic substitutability (BCG−). In this environment, own-strategy contemplation increases early at a lower sophistication level (IRT=−0.315), whereas gazing at opponents’ strategies lags significantly, increasing only after a higher sophistication level is attained (IRT=1.257), resulting in an expanded asymmetry delta of 1.571.

Taken together, these findings are consistent with a bounded-rationality account, offering suggestive evidence on its underlying cognitive architecture. Rather than a simultaneous expansion of informational capacity, strategic reasoning operates through a sequential hierarchy: individuals first secure a firm grasp on their own strategic options at lower-to-moderate levels of strategic sophistication, and only when they possess superior strategic sophistication do they formally internalize and model the strategic choices of their opponents.

**Result** **5.** *The estimated inflection thresholds are consistent with a non-linear progression in strategic engagement: at lower-to-moderate levels of strategic sophistication, players show increasing visual attention to their own strategies, consistent with best-response reasoning (MySt), while attention consistent with belief formation (OpSt) remains relatively flat; attention to OpSt rises only at higher levels of sophistication, with both measures elevated at the highest levels.*

A subject-level bootstrap of the difference between the two thresholds ($\Delta x^{*}=\;x_{MySt}^{*}-x_{OpSt}^{*}$) supports the direction of this pattern in both environments (point estimate $\Delta x^{*}\approx -0.81$ standard deviations of the sophistication score in each case), but the one-sided 95% confidence intervals do not exclude zero (BCG+: *p* = 0.062; BCG−: *p* = 0.162). We therefore treat this ordering as a suggestive, exploratory pattern rather than a statistically established sequence.

## 4. Discussion and Conclusions

In this paper we investigate how the strategic environment shapes the reasoning that precedes a choice, not only the choice itself. We used a within-subject design in which the same participants played a beauty contest game under strategic complementarity (BCG+) and under strategic substitutability (BCG−), and we recorded their eye movements throughout. Three results follow. First, we reproduced the strategic environment effect, with choices deviating more from the symmetric Nash equilibrium under complementarity than under substitutability. Second, the eye-movement record differed systematically across the two environments in a way that tracks the structure of the best-response correspondence. Participants visited the strategically critical regions of the payoff table more often and moved between them more frequently under substitutability, even though the overall number and duration of their fixations did not change. Third, strategic sophistication, measured by an item-response model estimated from diverse calibration tasks, was associated with more dynamic visual engagement across both environments. As subjects became more sophisticated, the two visual-attention measures associated with strategic reasoning strengthened in sequence rather than together, with attention to one’s own strategies rising before attention to the opponents’, and the ordering was the same in both environments. The rest of this section relates these findings to the experimental and eye-tracking literatures and considers their implications.

The strategic environment effect has been documented across many settings, from oligopoly experiments [1,5,20] to expectation-feedback markets [22,23,24,25] and guessing games [2,6]. In these lines of research, though, explanation attempts relied more on structural aspects. Hanaki et al. [6], for instance, show that the distribution of hierarchical-thinking levels is the same in the two environments, and that, holding this distribution fixed, iterated best responding necessarily produces wider dispersion around the equilibrium under complementarity than under substitutability. The reason is that, when actions are substitutes, a best response to others’ deviation lands on the opposite side of the equilibrium, so successive rounds of reasoning straddle the equilibrium and average toward it; when actions are complements, best responses stay on the same side and deviations accumulate. This account makes a sharp prediction about the reasoning process, but until now the prediction had been tested only through its imprint on choices.

Our data let us inspect that process. If subjects engage in the cross-equilibrium comparison that the structural account attributes to them, the gaze record under substitutability should show more movement between regions on opposite sides of the equilibrium, as is what we observe. Visit counts and, especially, fixation transitions are higher in BCG−, and the difference is concentrated in exactly the regions (LH and HL) where the best-response areas of the two games diverge. The pattern holds for vertical transitions, which we associate with best-response reasoning, and for horizontal transitions, which we associate with belief formation. The eye movements, thus, provide observable patterns consistent with a mechanism that earlier work only inferred, and they do so in a one-shot setting where explanations that rely on repeated interaction and learning (e.g., [5]) do not apply.

We argue, based on our data, that what differs across the two environments is the organization of gaze, not its amount. The total number of fixations and the total time spent looking at the payoff table are statistically indistinguishable between BCG+ and BCG−. How the gaze is allocated and how often it crosses between regions differs and the strategic environment reorganizes attention rather than increasing or decreasing it. This dissociation between the quantity of looking and its spatial organization is invisible to a choice-only analysis, and it suggests that the effort the environment elicits is better captured by the structure of visual search than by its overall volume.

Results from this study contribute to our understanding of the relationship between strategic sophistication and visual attention. We measure sophistication with an item-response model estimated from performance against computer opponents whose behavior corresponds to well-defined benchmarks (level-1, Nash, and inclusive cognitive hierarchy). Relative to the cognitive-reflection and fluid-intelligence measures used elsewhere [11,34,48,49], this index is incentivized, behavioral, and specific to strategic reasoning, and because it weights tasks by their difficulty it extracts more information from a small number of decisions than a raw score would.

The eye-movement data confirm and extend a robust regularity of the game-theoretic eye-tracking literature, namely that sophisticated players attend to both their own and their opponents’ incentives, whereas less sophisticated players neglect information about the counterpart [26,28,32,33,34]. In our experiment, both own-strategy and opponent-strategy attention rise with sophistication, in both environments. This carries the regularity from the small matrix games on which it was established to a guessing game with eleven pure strategies per player, which indicates that the link between sophistication and the breadth of strategic attention is not an artifact of the 2×2 and 3×3 formats.

A novel finding concerns the order in which visual attention related to the two components of reasoning becomes prominent as sophistication increases. Attention to one’s own strategies accelerates at a low level of sophistication, while attention to the opponents’ strategies accelerates only at a substantially higher level, and the gap between the two thresholds widens under substitutability. This sequence gives a pattern consistent with a process-level reading of the level-*k* and cognitive-hierarchy ladder [7,8,9]. The rungs differ in the depth of beliefs that players hold, and also in which part of the game they look at, with the corresponding pattern in gaze allocation running from securing one’s own best response to modeling the opponent. A similar ordering appears in studies that infer reasoning type from lookups in other classes of games, where sophisticated senders and learners are distinguished by their attention to others’ payoffs [50,51]. Part of becoming strategically sophisticated, then, is learning to attend to the opponent and not only to one’s own options.

Two features of the sophistication results should be read together. The mixed-effects estimates show that the association between sophistication and attentional intensity, measured by the number and duration of fixations, is the same in the two environments, with no interaction between sophistication and the environment. At the same time, the increase in gaze transitions under substitutability is largest for participants in the middle of the sophistication distribution. These two are consistent once one separates the volume of attention from its organization. How much a person looks is a stable feature of that person, well predicted by sophistication and unmoved by the environment. How that looking is organized into cross-region comparisons responds to the environment, and it does so most for the middle group. The least sophisticated lack the capacity to exploit the structure of either game, and the most sophisticated already search thoroughly in both, so it is the middle group that has the most room to respond to the extra comparisons substitutability calls for.

Methodologically, our study shows that eye-tracking tools can be fruitful in understanding strategic reasoning in games well beyond the small matrices to which the method has mostly been applied. Earlier work relied either on occluded-information interfaces, in which payoffs are hidden until a subject opens a box, in games from 2×2 to 4×4 [26,28,29,50,51], or on visible small matrices [30,31,32,33]. The closest precedent for process tracing in a guessing game is Costa-Gomes and Crawford [52], who applied the occluded-information method to two-person guessing games with large strategy spaces. Extending the open-display eye-tracking approach to a four-player guessing game with eleven pure strategies required three design choices. Namely, reducing the payoffs to a binary or ternary scale, shading the best-response cells so that subjects did not have to compute them, and defining areas of interest from theory so that they are designed to correspond to distinct reasoning operations. Together these choices strip away the information-acquisition and arithmetic stages and leave the reasoning stage, so that the gaze record is more likely to reflect the reasoning stage rather than these earlier stages. Our reading of vertical and horizontal transitions as best-response reasoning and belief formation follows a literature that links specific classes of gaze transitions to the decision rule a subject is applying [30,31,32,33,34]. The broader interpretation rests on the eye-mind hypothesis, that the locus of fixation indexes the locus of processing [44,45], on the classical finding that attention is allocated according to the goals of the task [53], and on evidence from value-based and consumer choice that fixations are tightly coupled to the construction of a decision [54,55,56]. Our results add the strategic environment to the list of task features that systematically shape where people look.

Several limitations qualify these conclusions and point to further work. Eye-tracking records overt attention, and the inference from gaze to reasoning, although grounded in the eye-mind hypothesis, is not direct. Covert attention, parafoveal processing, and reasoning from memory leave no fixation trace, and attention can shape choice as well as reflect it [54,56]. Furthermore, observed transitions and fixations may partly stem from non-cognitive or perceptual factors, such as perceptual scanning, visual comparison demands, interface geometry, or uncertainty, rather than pure iterative best-response computations. Our design choices that isolate the reasoning stage also limit external validity. By highlighting best responses and simplifying the payoffs, we removed the information-acquisition burden that is central to many applied settings, so what we observe may be closer to verification and comparison than to computation from scratch. The games are played once, which means we cannot relate to how the patterns we document evolve with experience, a question on which the literature on learning and on the plasticity of sophistication are informative [35,51]. Our sophistication index is estimated from six tasks and takes a limited number of values; the sample consists of university students drawn from a single subject pool, and the mapping from transitions to iterated best-response reasoning, though theoretically motivated, remains a proxy interpretation rather than a direct measurement. Extrapolation of our findings to richer field settings such as markets or negotiations remains a direction for future work. Finally, the areas of interest partition the strategy space coarsely, at the equilibrium; finer partitions and the joint elicitation of beliefs alongside gaze [31] would sharpen the link between what subjects look at and what they believe.

## Figures and Tables

**Table 1:** Summary of fixation count on the whole screen stratified by IRT score group.

Category	*N*	*BCG+*	*BCG*−	*p*-Value ^a^
fixation count (full sample)	86	52.4 (41.5)	56.8 (54.4)	0.798
*by IRT score*				
High	27	79.1 ^b^ (50.6)	71.4 ^e^ (67.8)	0.203
Mid	30	46.8 ^c^ (31.9)	68.4 ^e^ (55.4)	0.150
Low	29	33.5 ^d^ (26.6)	31.3 ^f^ (23.1)	0.681

Note: Standard deviations are reported in parentheses. ^a^ *p*-values are derived from within-subject paired Wilcoxon signed-rank tests comparing *BCG*+ and *BCG*−. ^b,c,d^ Means within the *BCG*+ column with different superscript letters significantly differ between IRT groups (*p* < 0.05 by unpaired Mann–Whitney U tests). Specifically, High > Mid (*p* = 0.0027) and Mid > Low (*p* = 0.0301). ^e,f^ Means within the *BCG*− column with different superscript letters significantly differ between IRT groups (*p* < 0.01 by unpaired Mann–Whitney U tests). Specifically, Mid > Low (p < 0.001), while High and Mid do not significantly differ (*p* = 0.8856).

**Table 2:** Average visit count and fixation duration within the payoff matrix.

	Visit Count			Fixation Duration (Sec.)		
	BCG+	BCG−	*p*-Value	BCG+	BCG−	*p*-Value
LL	8.05	8.35	0.301	5.24 ***	4.01	0.006
LH	2.09	4.42 ***	<0.001	0.54	1.44 ***	<0.001
HL	2.33	6.48 ***	<0.001	0.58	2.36 ***	<0.001
HH	3.15	3.21	0.669	1.24 *	1.11	0.071
Total (payoff area)	15.62	22.45 ***	0.008	7.59	8.92	0.969

Note: *N* = 86. *LL*, *LH*, *HL*, *HH* are the four areas of interest defined in Figure 3. Asterisks denote the significantly higher value within each environmental pair: *** significant at the 1% level, * at the 10% level. *p*-values are derived from Wilcoxon signed-rank tests.

**Table 3:** Within-subject gaze transitions across strategic environments stratified by strategic sophistication.

		Total Transitions		Vertical Transitions		Horizontal Transitions	
Group	*N*	BCG+	BCG−	BCG+	BCG−	BCG+	BCG−
full sample	86	8.47 (6.92)	15.06 (16.94)	3.26 (3.39)	6.28 (8.15)	2.92 (3.50)	7.01 (8.24)
*by IRT score*							
High	27	13.48 (9.25)	17.78 (18.00)	5.22 (4.64)	7.00 (9.43)	5.63 (4.78)	8.52 (9.25)
Mid	30	6.37 (3.60)	19.60 (20.15)	2.27 (1.82)	8.67 (9.15)	1.73 (1.39)	8.80 (9.64)
Low	29	5.97 (4.12)	7.83 (8.12)	2.45 (2.46)	3.14 (3.93)	1.62 (1.92)	3.76 (3.74)

Note: Standard deviations are reported in parentheses. *p*-values (two-tailed paired Wilcoxon signed-rank tests) for the full sample are *p* < 0.001 for all three metrics (*W* = 598.0, 809.5, 461.0 for total, vertical, and horizontal transitions, respectively).

**Table 4:** Linear mixed-effects estimates for fixation count and fixation duration.

	(1) Fixation Count		(2) Fixation Duration (s)	
Predictor	Coefficient	*p*-Value	Coefficient	*p*-Value
const.	52.371 *** (4.871)	<0.001	10.164 *** (0.986)	<0.001
IRTscore	23.009 *** (6.145)	<0.001	4.216 ** (1.244)	0.001
BCG−	4.409 (5.923)	0.457	1.211 (1.242)	0.330
IRTscore × BCG−	−0.620 (7.472)	0.934	−0.228 (1.567)	0.884
random effect (group var)	531.861		17.204	
log-likelihood	−884.020		−616.761	
no. observations (groups)	172 (86)		172 (86)	

Note: Standard errors are reported in parentheses. To account for within-subject repeated measurements, subject-specific random intercepts are estimated using a mixed-effects framework. ** *p* < 0.01, *** *p* < 0.001.

**Table 5:** Correlation analysis between strategic sophistication and visual attention.

		Pearson’s Correlation (*r*)		Spearman’s Rank Correlation (ρ)	
Environment	AOI Variable	Coefficient	*p*-Value	Coefficient	*p*-Value
BCG+	*MySt* (own)	0.275	0.010	0.333	0.002
(complementarity)	*OpSt* (opponent)	0.409	<0.001	0.463	<0.001
BCG−	*MySt* (own)	0.308	0.004	0.350	<0.001
(substitutability)	*OpSt* (opponent)	0.298	0.005	0.188	0.083

**Figure A1:**
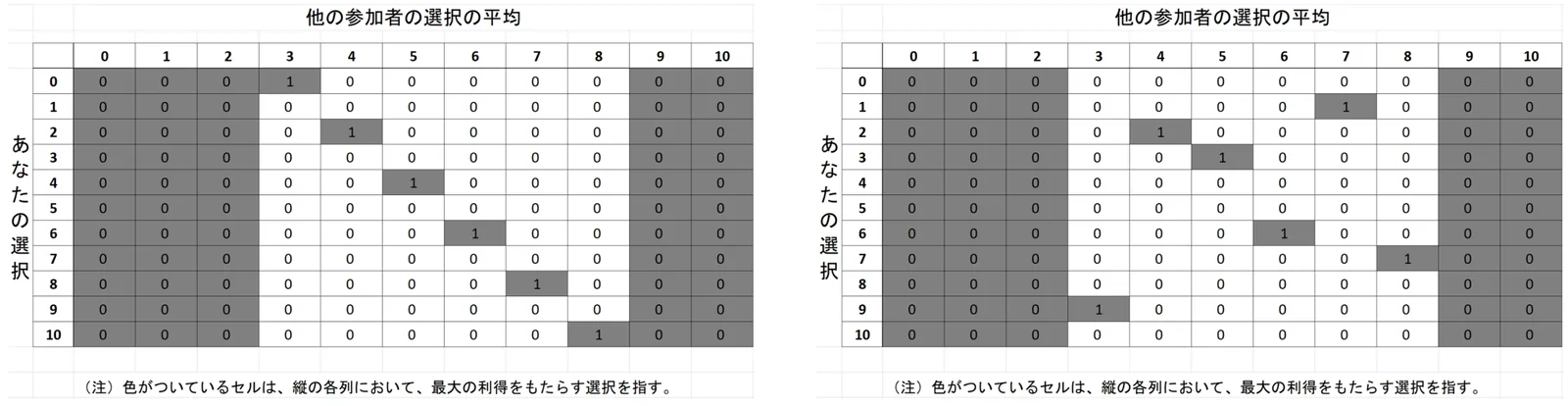
Payoff tables displayed on the screen during the Part 2 calibration tasks. Best-response regions are shaded. (**Left**): BCG1; (**Right**): BCG2.

## Data Availability

The data presented in this study are available on request from the authors. Raw gaze coordinates with high-resolution timestamps cannot be made publicly available, as their combination with device metadata may enable re-identification of participants, in violation of our ethics approval.

## Abbreviations

The following abbreviations are used in this manuscript:

AOI	Area of interest
BCG	Beauty contest game
DM	Decision maker
ICH	Inclusive cognitive hierarchy
IRT	Item response theory
NE	Nash equilibrium
SEE	Strategic environment effect

## Appendix A. Mixed-Effects and Within-Person Change Analysis Results

This appendix details the estimation results for both the level and within-person change analyses, which test the relationship between our process measures and behavioral outcomes (ChoiceDist, the absolute distance from the Nash equilibrium). Table A1 reports the linear mixed-effects regression estimates for decision outcomes and visual search metrics. Table A2 presents the within-person change (delta) analysis, examining how individual-level shifts in gaze behavior track changes in choice quality.

**Table A1:** Linear mixed-effects regressions on distance from Nash equilibrium.

	Dependent Variable: Choice Distance from NE (*ChoiceDist*)					
	(1)	(2)	(3)	(4)	(5)	(6)
Fixation Count	−0.0000					
	(0.0016)					
	[p=0.987]					
Fixation Duration		0.0018				
		(0.0080)				
		[p=0.824]				
Visit Count			−0.0010			
			(0.0043)			
			[p=0.812]			
Total Transitions				−0.0020		
				(0.0059)		
				[p=0.736]		
Vertical Transitions					−0.0067	
					(0.0119)	
					[p=0.577]	
Horizontal Transitions						−0.0000
						(0.0124)
						[p=0.999]
Environment (BCG−)	0.0701	0.0679	0.0768	0.0831	0.0901	0.0699
	(0.1200)	(0.1204)	(0.1231)	(0.1259)	(0.1250)	(0.1300)
	[p=0.559]	[p=0.573]	[p=0.533]	[p=0.510]	[p=0.471]	[p=0.590]
IRT Score	−0.3712 ***	−0.3789 ***	−0.3644 ***	−0.3627 ***	−0.3610 ***	−0.3721 ***
	(0.1171)	(0.1156)	(0.1156)	(0.1140)	(0.1126)	(0.1149)
	[p=0.002]	[p=0.001]	[p=0.002]	[p=0.001]	[p=0.001]	[p=0.001]
Constant	0.6074 ***	0.5849 ***	0.6288 ***	0.6360 ***	0.6473 ***	0.6062 ***
	(0.1417)	(0.1402)	(0.1438)	(0.1389)	(0.1299)	(0.1373)
	[*p* < 0.001]	[*p* < 0.001]	[*p* < 0.001]	[*p* < 0.001]	[*p* < 0.001]	[*p* < 0.001]
Random Effects (Group Var)	0.3588	0.3582	0.3582	0.3559	0.3580	0.3592

Standard errors in parentheses, *p*-values in brackets. *** *p* < 0.01.

**Table A2:** OLS regressions on within-person changes in equilibrium deviation (ΔChoiceDist).

	Dependent Variable: Within-Person Change in Equilibrium Deviation (ΔChoiceDist)					
	(1)	(2)	(3)	(4)	(5)	(6)
ΔFixation Count	0.0001					
	(0.0022)					
	[p=0.970]					
ΔFixation Duration		−0.0005				
		(0.0106)				
		[p=0.965]				
ΔVisit Count			0.0004			
			(0.0057)			
			[p=0.950]			
ΔTotal Transitions				0.0020		
				(0.0079)		
				[p=0.804]		
ΔVertical Transitions					−0.0057	
					(0.0156)	
					[p=0.716]	
ΔHorizontal Transitions						0.0117
						(0.0169)
						[p=0.489]
IRT Score	0.1002	0.1004	0.1010	0.1037	0.0970	0.1097
	(0.1517)	(0.1517)	(0.1521)	(0.1521)	(0.1518)	(0.1518)
	[p=0.510]	[p=0.510]	[p=0.508]	[p=0.497]	[p=0.524]	[p=0.472]
Constant	−0.0697	−0.0706	−0.0676	−0.0570	−0.0872	−0.0221
	(0.1206)	(0.1210)	(0.1264)	(0.1311)	(0.1290)	(0.1381)
	[p=0.565]	[p=0.561]	[p=0.594]	[p=0.665]	[p=0.501]	[p=0.873]

Standard errors in parentheses, *p*-values in brackets. All variables are calculated as $\Delta X=X_{\mathrm{BCG}+}-X_{\mathrm{BCG}-}$.

## Appendix B. Generalized Additive Model (GAM) Specification and Threshold Definition

Here we provide specification of the GAM used to capture potential non-linear relationship between subjects’ strategic sophistication ($x_{i}$, measured by the IRT score sophist) and their eye gaze metrics ($y_{i}$). Assuming a Gaussian error structure and an identity link function, the model is formulated as

$$y_{i}=\beta_{0}+f\left(x_{i}\right)+\varepsilon_{i},\;\varepsilon_{i}∼N\left(0,\sigma^{2}\right)$$

where $\beta_{0}$ is the intercept, $\varepsilon_{i}$ is the i.i.d. error term, and $f(x_{i})$ is a smooth, non-parametric function evaluated using penalized cubic B-splines with a basis dimension of k=10. The optimal smoothness parameter λ is determined endogenously via Generalized Cross-Validation (GCV) to minimize over-fitting.

We define the inflection threshold $x^{*}$ as the strategic sophistication level at which the eye gaze metric attains its maximum increase. Mathematically, $x^{*}$ is identified by computing the numerical first-order derivative of the fitted spline function f(x) over a grid of 200 evaluation points and locating its global maximum:

$$x^{*}=\arg\max_{x}\frac{df(x)}{dx}.$$

The uncertainty around the estimated function f(x) is evaluated using 95% Bayesian confidence intervals. Residual diagnostics, including quantile–quantile (Q-Q) plots and residual-versus-fitted checks, were conducted for each strategic specification to confirm homoscedasticity and normality assumptions.

## Appendix C. Design Details for Parts 1 and 2, and Experimental Instructions

This appendix provides the comprehensive parameter specifications, interface layouts, and procedural mechanics for the preparatory and trait-calibration phases of the experiment referenced in Section 2.4 of the main text.

### Appendix C.1. Part 1: General Familiarization

Part 1 was structured to eliminate initial learning noise and guarantee that all subjects understood the basic interaction under the *p*-Beauty Contest Games. In each game, participants selected an integer between 1 and 100 on the computer screen. The winner was defined as the participant whose chosen number was closest to the target number.

We implemented three variants of the target number:

$$\begin{array}{cc} \; & \mathrm{Game}\;G0:\;\frac{2}{3}x\\ \; & \mathrm{Game}\;G+:\;20+\frac{2}{3}x\\ \; & \mathrm{Game}\;G-:\;100-\frac{2}{3}x \end{array}$$

where *x* denotes the average of the numbers chosen by the other three participants. Game G0 corresponds to the standard Beauty Contest Game with a unique Nash equilibrium at the minimum under strategic complementarity. Games G+ and G− shift the unique Nash equilibrium to 60; however, G+ preserves strategic complementarity, whereas G− introduces strategic substitutability.

Subjects faced these games across three distinct settings regarding opponents’ behavior:

P1: Interacting with three human participants simultaneously playing in the other booths.
P2: Interacting with computerized opponents that select integers uniformly at random from 1 to 100.
P3: Interacting with computerized opponents that best respond to uniform random players.

Each subject completed a total of seven games in Part 1 (settings P1–P3 for both G+ and G−, and P2 for G0) in a randomized order.

### Appendix C.2. Part 2: Tasks for IRT Sophistication Score Elicitation

Part 2 served to elicit the subjects’ strategic sophistication. All subjects completed tasks using two structurally discrete games, BCG1 and BCG2, where the strategy set was restricted to integers from 0 to 10.

To maximize tractability and subjects’ familiarization, the payoff presentations were designed to be identical to those used in our main objective, Part 3, incorporating two key structural simplifications:

1. Payoffs depend on a player’s own strategy and the closest integer to the average of the other players’ choices, rather than non-integer averages.
2. A player wins the prize when her chosen integer is closest to her own target number, rather than comparing target distances globally across all players. If multiple players win, a fair lottery determines the final prize recipient.

As in Part 3, best-response cells were highlighted by shading to minimize the numerical computation burden (Figure A1). BCG2 functions as a strategically equivalent variant of BCG1, where the pure strategy labels x∈{0,1,…,10} are permuted by π, such that:

$${\left(\pi \left(x\right)\right)}_{x=0}^{10}=\left(2,0,1,4,7,8,5,3,10,9\right)$$

Under each game, subjects completed three tasks that systematically altered the behavior of automated computer opponents without providing feedback:

- Level-1 Task (L1): Opponents select each integer with equal probability.
- Nash Equilibrium Task (NE): Opponents choose the same number as the subject.
- Inclusive Cognitive Hierarchy Task (ICH): Opponents behave as in L1 with probability 50% and as in NE with probability 50%.

### Appendix C.3. Experimental Instructions of Part 3

The following instructions were displayed to subjects before they viewed the payoff table, and were used for both BCG+ and BCG−.

*You and three other participants will each choose a number between 0 and 10. Your payoff depends on the number you choose and the average of the numbers chosen by the other participants, as illustrated in the payoff table on the following screen. The exchange rate for your remuneration is 500 JPY per 1 point. Please carefully examine the payoff table on the next screen, and click on a number to make your choice. Once you understand these instructions, please click the screen to proceed.*

## Appendix D. Eye-Tracking and Experimental Settings

### Appendix D.1. Eye-Tracking Data Pre-Processing and Event-Detection Settings

Visual fixation events and area of interest (AOI) hit sequences were processed using the proprietary Tobii Fixation Filter in Tobii Studio. The filter identifies fixations by evaluating sample velocity and spatial dispersion. Specifically, gaze samples were parsed using a velocity threshold of 0.42 pixels/ms and a maximum spatial distance threshold of 35 pixels, requiring a minimum fixation duration of 16.6 ms. Data loss gaps shorter than 100 ms were linearly interpolated prior to event detection.

### Appendix D.2. Gaze Metrics and Operational Definitions

Visual search behavior was quantified using four distinct gaze metrics:

- Fixations: Identified using velocity- and distance-based thresholds. Fixations falling outside designated AOIs were retained as valid gaze events but categorized as non-AOI hits.
- AOI Hits: Fixations falling within the geometric boundaries of an AOI.
- Visits: Defined as a continuous sequence of one or more fixations within a specific AOI, bounded by entry into and exit from that AOI. Consecutive fixations landing inside the same AOI were merged into a single visit.
- Transitions: Saccadic movements between consecutive fixations exceeding the velocity threshold. All valid inter-AOI shifts, including repeated transitions between the same AOI pair, were counted.

AOI Boundary Sensitivity: To ensure robust signal capture without introducing spatial noise, we tested various buffer widths for the strategy labels (MySt and OpSt). The selected boundaries represent the optimal threshold balancing sample completeness and spatial accuracy.

### Appendix D.3. Eye-Tracking Calibration

Eye-tracking calibration was conducted using a standard 5-point calibration routine in Tobii Studio. Participants were seated at an optimal distance from the Tobii monitor such that both eyes were centrally aligned within the track status window. Before recording, participants were instructed to maintain a fixed posture and follow a moving red calibration dot across five screen coordinates. Upon completion of the initial sequence, calibration accuracy was visually evaluated; any points exhibiting poor accuracy or tracking failure were selectively recalibrated. If acceptable calibration quality could not be achieved after two to three recalibration attempts, the participant completed the session, but their gaze data were subsequently audited and excluded from analysis due to insufficient data quality.

### Appendix D.4. Apparatus and Signal Processing

Stimuli were presented on a 17-inch monitor with participants seated at a viewing distance of 50–80 cm, corresponding to a maximum gaze angle of approximately 35 degrees across the display area. Gaze coordinates were recorded binocularly and averaged across both eyes using Tobii Studio’s default algorithm. To handle temporary signal loss and blinks, missing data gaps were filled using linear interpolation performed independently on each eye’s data stream prior to event detection and AOI analysis.

### Appendix D.5. AOI Definition, Buffer and Overlap Rules

The explicit pixel coordinates and surface areas for the six primary AOIs used in our stimuli are defined as follows (origin (0,0) at top-left):

- LL: Vertices (108,105),(774,105),(774,378),(108,378); Area = 181,818 px.
- LH: Vertices (774,105),(1235,105),(1235,378),(774,378); Area = 125,853 px.
- HL: Vertices (108,378),(774,378),(774,567),(108,567); Area = 125,854 px.
- HH: Vertices (774,378),(1235,378),(1235,567),(774,567); Area = 87,129 px.
- MySt: Polygon vertices (20,106),(109,106),(161,130),(162,567),(18,567); Area = 65,078 px.
- OpSt: Polygon vertices (109,46),(109,106),(162,129),(1234,129),(1234,45); Area = 93,793 px.

Concerning the buffer sizes and overlap rules, no artificial padding buffers were added around the bounding coordinates for LL, LH, HL and HH, whereas buffers are added to MySt and OpSt, as illustrated in Figure 3 and Figure 5. While boundary intersections exist between certain payoff grid AOIs and the adjacent strategy label AOIs, this overlap does not introduce any measurement ambiguity or bias, since payoff cells and strategy labels are evaluated in separate, independent analyses, ensuring that a single fixation event is never double-counted within the same statistical model.

### Appendix D.6. Data Quality and Exclusion Criteria

Participant data quality was evaluated based on the proportion of valid gaze samples recorded by the eye-tracker. Following an a priori established protocol prior to data analysis, participants were excluded if they failed the criteria; out of 102 participants, 16 are excluded from the analysis. A total of 14 subjects whose total gaze rate falls below 50%, or whose gaze rate in either game falls below 10%, are excluded as these indicate insufficiently reliable eye-tracking data. Additionally, two subjects are excluded after inadvertently skipping the entire BCG− game with a mistaken click. Further information on exclusion criteria is provided in Appendix D. To verify that this exclusion scheme introduced no systematic bias, we compared baseline cognitive characteristics and behavioral choice patterns between the included and excluded samples. Two-sample *t*-tests revealed no statistically significant differences in estimated IRT strategic sophistication scores (t=0.075,p=0.941), average task performance (t=0.034,p=0.973), or specific equilibrium/heuristic choice frequencies (all p>0.24). These comparisons confirm that the quality-based exclusion protocol introduced no selection bias.

### Appendix D.7. IRT Model Specification and Parameters

Table A3 and Table A4 present the IRT model specification and parameters.

**Table A3:** Item parameters for the 2PL model (*N* = 102).

Item	Task Type	*p* (Correct)	Discrimination *a* (SE)	Difficulty *b* (SE)
L1KA	Level-1 (Game A)	0.363	0.468 (0.358)	−1.267 (1.007)
L1KB	Level-1 (Game B)	0.225	0.609 (0.466)	−2.184 (1.488)
NEKA	Nash equilibrium (Game A)	0.500	1.520 (0.746)	−0.000 (0.182)
NEKB	Nash equilibrium (Game B)	0.549	2.067 (0.965)	−0.160 (0.176)
ICHKA	Inclusive cognitive hierarchy (Game A)	0.686	1.432 (0.711)	−0.751 (0.355)
ICHKB	Inclusive cognitive hierarchy (Game B)	0.657	2.645 (1.206)	−0.484 (0.184)

Note: Standard errors are computed via a nonparametric bootstrap (300 resamples at the subject level). *p* (correct) denotes the proportion of subjects answering the item correctly.

**Table A4:** Sensitivity analyses for the strategic-sophistication measure (*N* = 102).

Comparison	Pearson’s *r*	Spearman’s ρ
6-item 2PL score vs. unweighted number correct	0.952	0.939
*leave-one-item-out: 5-item 2PL score (item omitted) vs. 6-item 2PL score*		
omit L1KA	0.994	0.995
omit L1KB	0.992	0.993
omit NEKA	0.960	0.947
omit NEKB	0.922	0.904
omit ICHKA	0.965	0.961
omit ICHKB	0.921	0.916

Note: All correlations are significant at *p* < 0.001. The unweighted score is the number of correct responses out of six tasks. Leave-one-item-out scores are obtained by re-estimating the 2PL model on the remaining five items and correlating the resulting ability estimates with the original six-item score.
